# Uptake of colorectal cancer screening after mailed fecal immunochemical test (FIT) outreach in a newly eligible 45–49-year-old community health center population

**DOI:** 10.1007/s10552-023-01717-8

**Published:** 2023-06-10

**Authors:** Meghan C. O’Leary, Daniel S. Reuland, Sara Y. Correa, Alexis A. Moore, Teri L. Malo, Xianming Tan, Catherine L. Rohweder, Stephanie B. Wheeler, Alison T. Brenner

**Affiliations:** 1 Lineberger Comprehensive Cancer Center, University of North Carolina at Chapel Hill, Chapel Hill, NC USA; 2 Department of Health Policy and Management, Gillings School of Global Public Health, University of North Carolina at Chapel Hill, Chapel Hill, NC USA; 3 Division of General Medicine and Clinical Epidemiology, University of North Carolina School of Medicine, Chapel Hill, NC USA; 4 Patient Support Pillar, American Cancer Society, Kennesaw, GA USA; 5 Department of Biostatistics, University of North Carolina at Chapel Hill, Chapel Hill, NC USA; 6 Center for Health Promotion and Disease Prevention, University of North Carolina at Chapel Hill, Chapel Hill, NC USA

**Keywords:** Colorectal cancer screening, Mass screening, Fecal immunochemical test, Primary health care, Randomized controlled trial, Community health centers

## Abstract

**Purpose:**

We assessed fecal immunochemical test (FIT) uptake following a mailed FIT intervention among 45–49-year-olds newly eligible for colorectal cancer (CRC) screening based on 2021 United States Preventive Services Task Force screening recommendations. We also tested the effect of an enhanced versus plain mailing envelope on FIT uptake.

**Methods:**

In February 2022 we mailed FITs to eligible 45–49-year-olds at one Federally Qualified Health Center (FQHC) clinic. We determined the proportion who completed FITs within 60 days. We also conducted a nested randomized trial comparing uptake using an enhanced envelope (padded with tracking label and colored messaging sticker) versus plain envelope. Finally, we determined the change in CRC screening by any modality (e.g., FIT, colonoscopy) among all clinic patients in this age group (i.e., clinic-level screening) between baseline and 6 months post-intervention.

**Results:**

We mailed FITs to 316 patients. Sample characteristics: 57% female, 58% non-Hispanic Black, and 50% commercially insured. Overall, 54/316 (17.1%) returned a FIT within 60 days, including 34/158 (21.5%) patients in the enhanced envelope arm versus 20/158 (12.7%) in the plain envelope arm (difference 8.9 percentage points, 95% CI: 0.6–17.2). Clinic-level screening among all 45–49-year-olds increased 16.6 percentage points (95% CI: 10.9–22.3), from 26.7% at baseline to 43.3% at 6 months.

**Conclusion:**

CRC screening appeared to increase following a mailed FIT intervention among diverse FQHC patients aged 45–49. Larger studies are needed to assess acceptability and completion of CRC screening in this younger population. Visually appealing mailers may improve uptake when implementing mailed interventions.

*Trial registration* The trial was registered on May 28, 2020 at ClinicalTrials.gov (identifier NCT04406714).

## Background

In May 2021, the U.S. Preventive Services Task Force (USPSTF) extended colorectal cancer (CRC) screening recommendations to adults aged 45–49 who are at average risk for CRC [1]. The USPSTF assigned a “B” recommendation to this change, indicating there is moderate certainty that CRC screening in the 45–49-year-old population will have moderate net benefit, compared to its “A” recommendation for CRC screening among adults aged 50–75. They also called for additional research focused on screening effectiveness in the younger age group [1].

Mailing at-home CRC screening tests, including fecal immunochemical tests (FIT), directly to patients has potential to promote uptake of CRC screening in this newly eligible population of 45–49-year-olds. In addition to promoting FIT uptake, these programs may serve as general reminders about CRC screening and induce screening by other types of screening tests (e.g., colonoscopy). Mailed FIT outreach has been shown to be an effective evidence-based intervention that is associated with increased CRC screening compared to usual care among the 50–75-year-old population [2–5]. For example, in a meta-analysis, Jager and colleagues (2019) found a 2.65-fold increased likelihood of CRC screening completion with mailed stool testing-based outreach programs compared to usual care [2]. Furthermore, mailing stool kits to patients’ homes has been an important strategy for improving CRC screening in communities with high CRC burden, low screening rates, and/or for whom access to care may be limited [2, 3, 6–10].

Research has yet to focus specifically on the effectiveness of mailed FIT programs in the 45–49-year-old population, especially in more resource-limited settings such as Federally Qualified Health Centers (FQHCs) which receive federal funds to serve medically underserved areas and populations regardless of patients’ ability to pay for care. This will be particularly important to understand as FQHCs may soon be required to report CRC screening in this younger age category as a Uniform Data System (UDS) measure and, thus, need to prioritize achieving high screening rates in this population. In addition, little is known about how modifying the appearance of mailed outreach materials may affect uptake of these mailed FIT interventions. Gupta and colleagues (2020) reported that a common challenge with mailed FIT programs is getting patients to open the envelopes, with patients often not remembering that they received a letter or FIT packet [4]. While they recommended using more “eye-catching” materials to improve the likelihood that patients will open and respond to these mailings, different approaches to improving the outward appearance of mailed FIT envelopes have not been tested.

Therefore, in addition to assessing FIT uptake (effectiveness) associated with an organized mailed FIT outreach (hereafter “mailed FIT”) intervention among 45–49-year-old patients at a single FQHC clinic site, we also evaluated the effect of using an enhanced envelope (i.e., FIT mailed in a padded envelope with a tracking label and messaging sticker) versus a plain envelope on mailed FIT uptake in this population. Finally, in order to evaluate the ability of these mailed FITs to serve as broad screening reminders and to prepare for future reporting of CRC screening in this age category, we determined the change in clinic-level CRC screening, i.e., the proportion of 45–49-year-old patients at this FQHC who were up-to-date on CRC screening by any modality recommended by the USPSTF (e.g., FIT, colonoscopy), between baseline and 6 months after the mailed FIT intervention.

## Methods

Building on an ongoing academic/community partnership and research study, we implemented an expansion of a mailed FIT intervention to include the newly eligible age group of 45–49-year-old patients at a single FQHC clinic site in North Carolina. The intervention tested in the parent study, Scaling Colorectal Cancer Screening Through Outreach, Referral, and Engagement (SCORE), included centralized mailed FIT outreach and, for those with abnormal FIT results, patient navigation to follow-up colonoscopy; the parent study has been previously described [11]. Expanding the existing study protocol to this age group was feasible for several reasons: (1) moving from concept to implementation was rapid because few changes had to be made to the intervention protocol of the parent study and (2) the FQHC clinic supported targeting patients newly eligible for CRC screening in anticipation of changes to their UDS reporting requirements and benefitting their future funding streams.

The SCORE parent study and sub-study were conducted as part of the NCI-funded Accelerating Colorectal Cancer Screening and Follow-up through Implementation Science (ACCSIS) Consortium. The overall aim of the ACCSIS Consortium is to conduct multi-site, coordinated, transdisciplinary research to evaluate and improve CRC screening processes using implementation science. The sub-study was registered on ClinicalTrials.gov (NCT04406714) and approved by the University of North Carolina at Chapel Hill Institutional Review Board.

### Intervention description

Briefly, the mailed FIT intervention consisted of an introductory letter notifying patients that they would be receiving a FIT in the mail, followed by a mailed FIT kit and up to two mailed reminders. This intervention, described in detail elsewhere [11], was developed for low health literacy populations and is designed to be accessible to both English- and Spanish-speaking populations as well as to insured and uninsured patients. We adapted materials from the parent study and tailored them to include language about CRC screening for patients aged 45–49 years. Adaptations included reference to a change in the CRC screening recommendations, broadening age eligibility from age 50 down to age 45, and reasons why screening is being expanded to include this new, younger age group.

The mailed FIT outreach activities were performed by a team working at an academic cancer center on behalf of and in coordination with staff at the FQHC clinic. FIT kits were mailed in February 2022 to eligible patients. The FITs were mailed and processed at no cost to the patient. The team also provided phone-based patient navigation to support patients with an abnormal FIT result in the completion of a follow-up colonoscopy. The patient navigation component was included because patients are not considered up-to-date with CRC screening unless they complete a follow-up colonoscopy after an abnormal FIT. As in the parent study, patient navigation focused on assessing and addressing patients’ financial, transportation, emotional, and informational barriers to colonoscopy [11].

### Eligibility

We used an electronic medical record (EMR) query, augmented by a brief manual chart scrub, to identify all patients who were in the 45–49-year-old age group as of February 2022, had a primary care visit at the FQHC clinic in the prior 18 months (i.e., likely to be active patients per input from the clinic team), were at average risk for CRC, had a North Carolina address, and were not up-to-date on CRC screening. The query and patient eligibility assessment protocols were identical to those used in the larger trial [11]; a clinic representative ran a pre-built report including all active 45–49-year-old patients and study team members excluded patients with exclusionary criteria either shown in the report itself or through a brief, manual chart review (“scrub”). Being up-to-date on CRC screening was defined as having completed a colonoscopy in the past ten years, sigmoidoscopy, computerized tomography (CT) colonography or barium enema in the past five years, FIT DNA test in the past three years, or a FIT or fecal occult blood test in the past year.

**Fig. 1:**
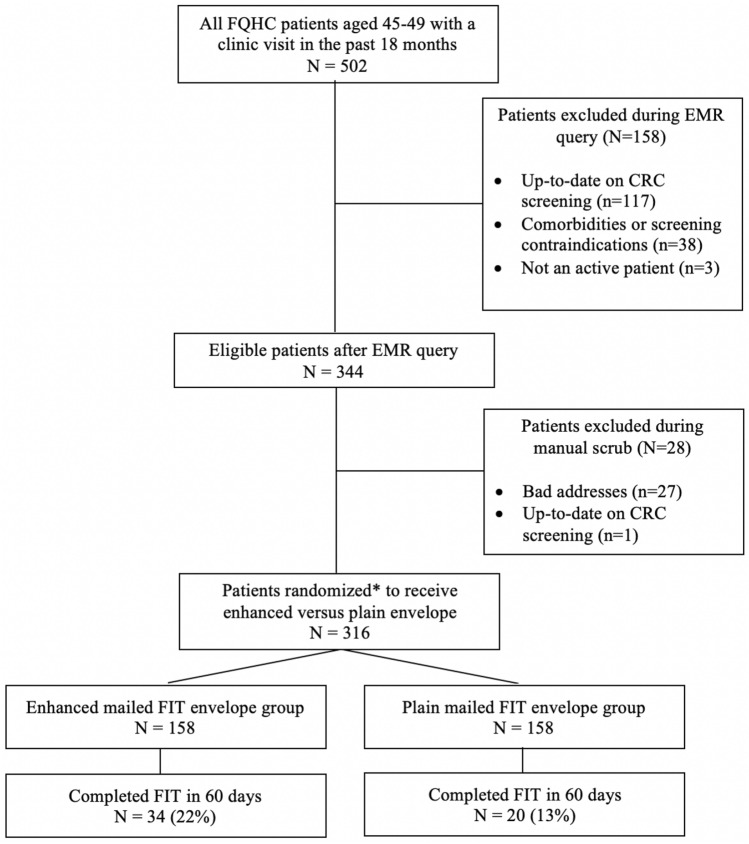
Consort diagram. *This was an intention-to-treat analysis

Patients were excluded from this sub-study if they were known to be at increased risk for CRC due to a personal history of CRC, defined as documented CRC, colorectal polyps, or colonic adenomas; family history of CRC among a first degree relative; or a diagnosis of inflammatory bowel disease. National guidelines do not recommend screening for individuals with limited life expectancy [12, 13]. Therefore, other exclusions included documented comorbidities and conditions associated with limited life expectancy (dementia, hospice care, assisted living, end-stage renal disease, glioblastoma, pancreatic cancer, lung cancer, esophageal cancer, liver and bile duct cancer, and mesothelioma). Following the electronic query, we conducted a manual EMR “scrub” to confirm the accuracy of the query and excluded additional patients with incorrect addresses or relevant health history (e.g., recent CRC screening) not captured by the query. Although EMR data do not perfectly represent CRC screening activities, these records reflected the most complete information available to providers at this site. Figure 1 provides a CONSORT flow diagram that summarizes the inclusion and exclusion criteria for this population.

### Randomization

**Fig. 2:**
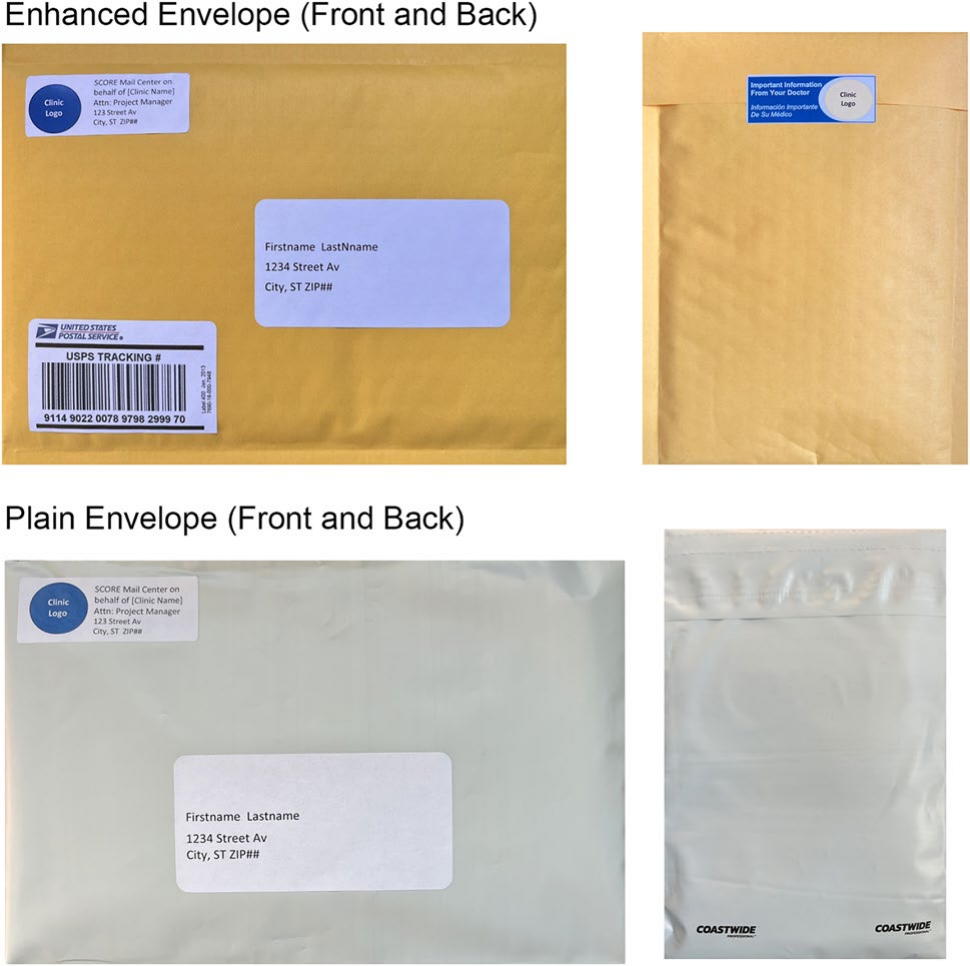
Enhanced and plain mailed FIT envelopes used in the mailed FIT nested randomized trial

As part of this sub-study, we also conducted a nested trial in which patients were randomized to one of two intervention arms to test the effectiveness of using an enhanced mailed FIT envelope versus a plain mailed FIT envelope. As shown in Fig. 2, the enhanced envelope was a padded yellow envelope that contained a U.S. Postal Service tracking label as well as a colored sticker with the message “Important information from your doctor.” In contrast, the plain white envelope did not have padding, a tracking label, or a message sticker.

We randomly assigned patients 1:1 to receive the enhanced or plain envelope. Using a computerized random number generator, we randomly sorted the eligible patient list and assigned one-half to the plain envelope and the other to the enhanced envelope. All other intervention characteristics were the same for patients in both intervention arms of the nested trial; the only difference was the type of mailed envelope used.

### Outcomes

Mailed FIT uptake: The primary outcome assessed was mailed FIT uptake (effectiveness) within 60 days of randomization among 45–49-year-olds who were eligible for the intervention. Secondarily, we assessed FIT uptake within 6 months of randomization in this group. The 60-day and 6-month endpoints were established a priori*.* In addition, we assessed differences in FIT uptake during this timeframe by demographic factors (sex, race/ethnicity, and insurance status as reported in the EMR), as well as by exposure to the enhanced versus plain mailed FIT envelope. FIT uptake was determined using records from the laboratory that processed all SCORE FIT kits. We also assessed completion of follow-up colonoscopies among patients who had abnormal FITs using navigator case management logs.

Overall clinic-level CRC screening: To understand how this intervention might affect overall screening among 45–49-year-olds at this clinic (i.e., an endpoint that might eventually be a required UDS reporting metric by an FQHC), we compared the proportion of patients in this age group who were up-to-date with CRC screening between baseline and 6 months after the mailed FIT intervention at this site. In this observational component of our study, we determined clinic-level CRC screening using an EMR query to assess screening among all patients at this site in the 45–49-year-old age category, including those eligible to receive the mailed FIT intervention and those who were ineligible because of risk factors (such as inflammatory bowel disease) that make colonoscopy the preferred initial screening test. We identified patients meeting this broader eligibility criterion and assessed their screening at two timepoints (baseline, 6-month post-intervention), allowing for patients to move in and out of this cohort (i.e., the total number of patients varied between timepoints). We report the proportion of patients who were current with recommended screening by any CRC screening test, including having had a colonoscopy in the past ten years, sigmoidoscopy, CT colonography or barium enema in the past five years, FIT DNA test in the past three years, or a stool test in the past year, per the clinic’s EMR.

### Statistical analysis

To assess effectiveness of the intervention, we calculated mailed FIT return at 60 days and 6 months. For the nested trial, we conducted an intention-to-treat analysis (i.e., all participants randomized included in the analysis). To compare FIT return between the two nested intervention arms (enhanced vs. plain envelope), we used a chi-squared test and calculated confidence intervals. We also used logistic regression to adjust for baseline demographic characteristics (sex, race/ethnicity, insurance). Finally, we calculated the difference in overall CRC screening at the FQHC clinic site in a 6-month period (February–August 2022)—accounting for all CRC screening modalities and measured using EMR data—and noted the types of modalities used. All tests were 2-sided at alpha level 0.05 unless otherwise specified. Statistical analyses were conducted using Stata software, version 17.0 (StataCorp, College Station, TX).

## Results

A total of 502 patients aged 45–49 had a clinic visit at this site in the past 18 months at baseline. Upon further review, we identified 316 of these patients who were eligible for the mailed FIT intervention study (Fig. 1). Table 1 describes the demographic and insurance characteristics of the 316 patients who were mailed a FIT kit, overall and by intervention arm. In the overall population, 57% were female, 58% were non-Hispanic Black, 27% were non-Hispanic White, and 8% were Hispanic. Participants varied with respect to insurance status (50% commercially insured, 29% uninsured, 13% Medicaid enrollees, and 8% Medicare enrollees).

**Table 1:** Characteristics of patients aged 45–49 who received the mailed FIT intervention, overall and by intervention arm

Characteristic	All (*n* = 316)	Enhanced envelope (*n* = 158)	Plain envelope (*n* = 158)
Sex			
Female	181 (57%)	84 (53%)	97 (61%)
Male	135 (43%)	74 (47%)	61 (39%)
Race/ethnicity			
Non-Hispanic white	84 (27%)	47 (30%)	37 (23%)
Non-Hispanic black	184 (58%)	88 (56%)	96 (61%)
Hispanic	25 (8%)	13 (8%)	12 (8%)
Other/unknown	23 (7%)	10 (6%)	13 (8%)
Insurance type^a^			
Commercial	159 (50%)	83 (53%)	76 (48%)
Medicaid	41 (13%)	18 (11%)	23 (15%)
Medicare	24 (8%)	15 (9%)	9 (6%)
Self-pay/uninsured	91 (29%)	42 (27%)	49 (31%)

^a^Insurance status was not available in the EMR for one patient

Mailed FIT Uptake: Among the 316 patients who were mailed a FIT as part of the intervention, 54 (17%) returned a mailed FIT within 60 days and 3 additional patients returned a mailed FIT between 60-days and 6-months post-FIT mailing, for a total return of 18% in the 6-month period (Table 2).

**Table 2:** Proportion of patients aged 45–49 who returned a mailed FIT within 60 days post-FIT mailing

Characteristic	All screened/total (%)	Enhanced envelope screened/total (%)	Plain envelope screened/total (%)	Difference* (95% CI)
Proportion screened	**54/316 (17.1)**	**34/158 (21.5)**	**20/158 (12.7)**	**8.9 (0.6, 17.1)**
Sex				
Female	29/181 (16.0)	18/84 (21.4)	11/97 (11.3)	10.1 (−0.7, 20.9)
Male	25/135 (18.5)	16/74 (21.6)	9/61 (14.8)	6.9 (−6.1, 19.8)
Race/ethnicity				
Non-Hispanic white	16/84 (19.1)	10/47 (21.3)	6/37 (16.2)	5.1 (−11.6, 21.7)
Non-Hispanic black	31/184 (16.9)	21/88 (23.9)	10/96 (10.4)	**13.4 (2.6, 24.2)**
Hispanic	5/25 (20.0)	3/13 (23.1)	2/12 (16.7)	6.4 (−24.7, 37.5)
Other/unknown	2/23 (8.7)	0/10 (0)	2/13 (15.4)	−15.4 (−35.0, 4.2)
Insurance Type				
Commercial	28/159 (17.6)	19/83 (22.9)	9/76 (11.8)	11.0 (−0.5, 22.6)
Medicaid	9/41 (22.0)	5/18 (27.8)	4/23 (17.4)	10.4 (−15.5, 36.2)
Medicare	3/24 (12.5)	2/15 (13.3)	1/9 (11.1)	2.2 (−24.6, 29.0)
Self-pay/uninsured	14/91 (15.4)	8/42 (19.1)	6/49 (12.2)	6.8 (−8.2, 21.8)

*Bold text represents statistically significant findings for unadjusted differences between arms. In the logistic regression model controlling for all covariables, only type of envelope was found to be statistically significant (*p* < .05)

By study arm, 34/158 (22%) patients who received the enhanced envelope returned a FIT within 60 days, compared to 20/158 (13%) who received the plain envelope (difference 8.9 percentage points, 95% CI 0.6, 17.1; *p* = 0.04). In a post hoc logistic regression model controlling for demographic variables, only type of envelope was found to be statistically significant (*p* < 0.05).

Receipt of Follow-Up Colonoscopy: Of the patients who returned a mailed FIT within 60 days, 3/54 (6%) had an abnormal result—two of these patients were in the enhanced envelope arm and one was in the plain envelope arm. All three patients were successfully contacted by phone by the patient navigator and completed at least one navigation call. Additionally, all three patients completed a follow-up colonoscopy within 60 days of being notified about their abnormal FIT result. Of the three additional patients who returned a mailed FIT between 60 days and 6 months post-intervention, none had an abnormal FIT.

Overall Clinic-Level CRC Screening: The proportion of the clinic’s 45–49-year-old population that was up-to-date on CRC screening (across all CRC screening modalities) at baseline was 26.7% (134/502 patients). This larger population cohort included those who received the mailed FIT intervention, as well as other 45–49-year-old patients at the clinic who did not receive the intervention because they did not meet the more specific eligibility criteria for the mailed FIT intervention. Six months after the mailed FIT intervention, the proportion screened in this cohort was 43.3% (234/540 patients) and a difference of 16.6 percentage points (95% CI 10.9, 22.3). In terms of screening modalities used, at baseline, 24 (17.9%) of the screened patients were up-to-date by a stool test alone, 104 (77.6%) by a colonoscopy alone, and 6 (4.5%) by both a stool test and colonoscopy. In contrast, six months post-intervention, 72 (30.8%) patients screened were up-to-date by stool testing alone (which could include FITs received as part of the intervention or other stool-based screening tests), 145 (62.0%) by colonoscopy alone, and 17 (7.3%) by both stool testing and colonoscopy. Of the 234 patients up-to-date on screening at 6 months, 98 (41.9%) had received the mailed FIT intervention.

## Discussion

This study found that 17.1% of diverse FQHC patients aged 45–49 who received a mailed FIT intervention returned a FIT within 60 days and that the type of mailed FIT envelope used mattered (21.5% for enhanced vs. 12.7% for plain envelope). The observed increase in the proportion of all clinic patients in this age group who were up-to-date on CRC screening over a 6-month period, during which we implemented the mailed FIT intervention, suggests that there are opportunities to use a mailed FIT intervention as a strategy to support adherence to the updated CRC screening recommendation in this newly eligible age group.

We found that mailing FITs in an enhanced envelope was associated with improved CRC screening compared to mailing FITs in a plain envelope in this population, suggesting that there may be benefits to using more visually appealing mail packaging materials when implementing mailed interventions. We are unaware of other studies currently testing the impact of modifying the outward appearance of mailed FIT materials on uptake. Since our nested randomized trial was conducted at a single FQHC clinic site, we encourage further research in this area to determine packaging characteristics that are most associated with mailed FIT uptake. For example, it might be helpful to conduct interviews and focus groups with patients aged 45–49 about their preferred characteristics of mailed interventions. It is important to note that our enhanced mailing envelope was inexpensive yet associated with a statistically significant increase in FIT screening. Given the low cost, utilizing an enhanced mailing envelope is likely a feasible option for other implementation teams to adopt in order to boost CRC screening. Additionally, we found that nearly all patients who returned a mailed FIT did so within 60 days of the FIT mailing, resembling findings from prior research [14] and suggesting that mailed interventions are beneficial in terms of promoting prompt uptake of CRC screening.

Our study provided some early insight into the general uptake of CRC screening, especially FIT, among 45–49-year-olds. Forty-three percent of clinic patients in this age category were up-to-date on CRC screening 6 months after our mailed FIT intervention. Given that the USPSTF screening recommendation was updated in May 2021, there are limited published data on response rates to CRC screening interventions in other 45–49-year-old populations. Perhaps not surprisingly, FIT uptake in this younger age group following our mailed intervention appeared to be lower (though we did not have a control arm) than mailed FIT uptake previously observed in adults aged 50–75 [2, 5]. Based on 2021 UDS data for this FQHC, 59% of clinic patients aged 50–75 were up-to-date on CRC screening [15]. Issaka and colleagues (2019) reported that, across ten randomized studies, mailed FIT outreach in 50–75 years old was associated with median improvement in CRC screening of 21.5% (interquartile range: 13.6–29.0%) compared to controls [5]. The difference between age categories may be due, in part, to increased awareness about the recommendation for CRC screening for adults aged 50–75, whereas additional efforts are likely needed to widely communicate the screening recommendation change and promote CRC screening in the 45–49-year-old population. For example, most patients in the 45–49 age category have likely not received a recommendation from their primary care provider to complete CRC screening. Furthermore, healthcare system changes to support delivery of screening in new populations, such as EMR reprogramming, insurance reimbursement, and changes in screening-related quality metrics, take time to implement.

In our case, we were able to leverage an existing academic/community partnership and established screening intervention protocol used in our parent study to quickly respond to the updated screening recommendations. Having an existing partnership structure and protocol in place allowed us to begin mailing FIT kits to adults aged 45–49 within nine months of the recommendation change and to offer patient navigation to follow-up colonoscopy for patients with an abnormal FIT result. Based on prior trends in response rates to screening recommendation changes [16–18], we felt it was critical to use this opportunity to help accelerate CRC screening in this younger FQHC population. Horn and Haas underscored the need for well-designed screening support programs, along with policy and regulatory changes, to avoid unintended consequences, such as increased disparities in screening rates, following the USPSTF CRC screening recommendation change [19]. We similarly anticipate possible lags in CRC screening among 45–49-year-olds in low-income and medically underserved populations, such as those served by FQHCs, compared to more well-resourced communities. Implementation of mailed FIT interventions that prioritize reaching historically underserved populations in this younger age group may be an effective way of providing additional opportunities to provide CRC screening education and promotion in addition to visit-based screening discussions.

Mailed FIT outreach interventions may be particularly helpful for improving CRC screening among adults in the 45–49 age category for a few reasons. Individuals in this age group likely have less awareness that they are eligible for CRC screening. In addition, this age group tends to have fewer chronic conditions, compared to the older age group recommended for CRC screening and therefore attends primary care visits less frequently. Older patients and those with chronic conditions, such as diabetes, are more likely to screen for CRC compared to their counterparts [20, 21], presumably because they are more likely to visit the doctor and, thus, have additional opportunities to discuss screening with their provider. Prior research has shown that individuals without a usual source of care or who have not visited the doctor in the past year are less likely to be up-to-date on cancer screening than their counterparts [22], indicating that mailed interventions may be needed to reach these individuals instead. Thus, the 45–49-year-old population may be uniquely positioned to benefit from education about and complete screening through mailed outreach instead of in-person clinic visits.

This study has notable strengths. To our knowledge, it is among the first to report findings from a mailed FIT intervention targeted to patients aged 45–49 years and to test the effect of using an enhanced versus plain envelope in increasing uptake. This study also has limitations. First, this was a small study conducted at a single FQHC clinic site and, therefore, the results may not be generalizable to populations served by other clinics and health systems. Our goal was to provide some early estimates of mailed FIT uptake in the newly age-eligible population. Larger studies of interventions to promote CRC screening in this younger age category, as well as randomized studies with a control arm, are needed to support adoption and adherence to the revised screening recommendation. Second, we used an EMR query to assess overall clinic-level CRC screening pre- and post-intervention, which can include incomplete or inaccurate information about the receipt and results of CRC screening tests. Third, while we were able to randomize patients to receive different types of mailed FIT envelopes, we did not have a control arm, and the change in clinic-level screening over six months was based on observational data only. The trends we observed may, therefore, have been influenced by other factors such as increased awareness among patients and providers about 45–49-year-olds being newly recommended for CRC screening and changing patterns in CRC screening over time following the height of the COVID-19 pandemic. Fourth, we included patients targeted in the mailed FIT outreach in the final calculations of clinic-level screening at six months regardless of whether they appeared in the automated query. We did this because we considered them to be active patients since we were intervening clinically; however, this may have slightly overestimated the proportion screened in the strictly defined active patient population based on a face-to-face visit requirement. Lastly, we were only able to compare two different types of mailed FIT envelopes in our nested trial. Although we found a positive effect of using our enhanced mailing envelope compared to the plain envelope, further testing is needed to better understand the specific features of mailed materials associated with increased screening.

In conclusion, our study suggests that mailed FIT outreach could be used to accelerate the uptake of CRC screening in the newly eligible age group of 45–49-year-old patients. Identifying opportunities to increase the effectiveness of these interventions in this younger population will be important to ensure equitable screening outcomes in response to the updated screening recommendation.

## Data Availability

The datasets used during the current study are available at https://dataverse.unc.edu/dataverse/CCSI-SCORE.
